# Sensory stimulation-based protection from impending stroke following MCA occlusion is correlated with desynchronization of widespread spontaneous local field potentials

**DOI:** 10.1038/s41598-022-05604-1

**Published:** 2022-02-02

**Authors:** Waqas Rasheed, Anirudh Wodeyar, Ramesh Srinivasan, Ron D. Frostig

**Affiliations:** 1 Department of Neurobiology and Behavior, University of California, Irvine, CA USA; 2 Center for the Neurobiology of Learning and Memory, University of California, Irvine, CA USA; 3 Department of Cognitive Science, University of California, Irvine, CA USA; 4 Department of Statistics, University of California, Irvine, CA USA; 5 Department of Biomedical Engineering, University of California, Irvine, CA USA; 6 Department of Mathematics and Statistics, Boston University, Boston, MA USA

**Keywords:** Neuroscience, Medical research

## Abstract

In a rat model of ischemic stroke by permanent occlusion of the medial cerebral artery (pMCAo), we have demonstrated using continuous recordings by microelectrode array at the depth of the ischemic territory that there is an immediate wide-spread increase in spontaneous local field potential synchrony following pMCAo that was correlated with ischemic stroke damage, but such increase was not seen in control sham-surgery rats. We further found that the underpinning source of the synchrony increase is intermittent bursts of low multi-frequency oscillations. Here we show that such increase in spontaneous LFP synchrony after pMCAo can be reduced to pre-pMCAo baseline level by delivering early (immediately after pMCAo) protective sensory stimulation that reduced the underpinning bursts. However, the delivery of a late (3 h after pMCAo) destructive sensory stimulation had no influence on the elevated LFP synchrony and its underpinning bursts. Histology confirmed both protection for the early stimulation group and an infarct for the late stimulation group. These findings highlight the unexpected importance of spontaneous LFP and its synchrony as a predictive correlate of cerebral protection or stroke infarct during the hyperacute state following pMCAo and the potential clinical relevance of stimulation to reduce EEG synchrony in acute stroke.

## Introduction

Stroke is the fifth leading cause of death in the United States and a leading cause for long-term disability, and most cases are due to ischemia^1^. We have used a rat model of cerebral ischemia, a dorsal permanent Middle Cerebral Artery occlusion (pMCAo) that results in cortical infarct and functional deficits 24 h after ischemic onset as assessed by functional imaging, blood flow imaging, neuronal recordings, and histological staining, in addition to behavioral impairments assessed one-week post occlusion^2^. However, applying the same assessment techniques we have demonstrated that intermittent sensory (whisker) stimulation delivered during the hyperacute state post pMCAo as an ‘early’ treatment immediately after pMCAo (+0 h), 1 h or 2 h after pMCAo (+1 h and +2 h respectively) protects the cortex from ischemic stroke damage (see similar findings^3,4^). Beyond the 2 h early protection window, the same sensory stimulation treatment is not protective anymore and could even exacerbate damage if delivered as ‘late’ treatment 3 h (+3 h) after ischemic onset (reviewed in^5^).

Analyzing neuronal activity dynamics within the ischemic area may enable identification of pathophysiological characteristics post-pMCAo. Indeed, our previous study, using a 32-microelectrode array spanning depths of primary somatosensory cortex (S1) and neighboring cortical regions, provided a unique, continuous spatiotemporal profile of either spontaneous or evoked local field potentials (LFPs) and evoked multi-unit activity (MUA) that were recorded from the MCA territory before pMCAo (baseline) and during the hyperacute post-ischemic (0–5 h) period^6^. This study revealed that during the post-pMCAo recording period evoked MUA responses didn’t change compared to baseline whereas evoked LFP responses changed only at 3–5 h following pMCAo. However, unlike the evoked responses, when the no-stimulation control group was continuously recorded before and 5 h following pMCAo, spontaneous LFPs spatiotemporal synchrony increased significantly within minutes after pMCAo in the entire recording volume (4.5 mm distance between the first and last microelectrode, 1.5 mm depth). This synchrony remained high during the rest of the recording period, resulting in a clear infarct as revealed in the post-mortem histology. The widespread increase in spontaneous LFPs’ spatiotemporal synchrony and the presence of an infarct were not seen in a control no-stimulation surgical sham group. Further analysis revealed that the underlying source of such post-pMCAo widespread spontaneous LFP spatiotemporal synchrony was an increase in synchrony of intermittent bursts of low multi-frequency oscillations^6^.

The unexpected widespread synchronization of spontaneous LFPs following pMCAo and their underlying intermittent bursts prompted us to further study spontaneous LFP dynamics in other experimental pMCAo groups. In the current study, employing the same microelectrode array, we investigated the behavior of hyperacute post-ischemic spontaneous LFP dynamics in protected (early +0 h stimulation) and non-protected (late +3 h stimulation) sensory stimulation treatment groups in addition to relevant results obtained from a surgical sham group. We replicated our previous findings regarding the significant widespread increase in spontaneous LFPs spatiotemporal synchrony following pMCAo in both sensory treatment groups. However, enhanced post-ischemic LFP spatiotemporal synchrony is reduced to pre-pMCAo baseline level during protective early sensory stimulation treatment, but remained elevated throughout the damaging late stimulation. Intermittent low multi-frequency spatiotemporal bursts were the underlying source of synchrony in both experimental groups. Spatiotemporal synchrony results are consistent with infarct presence as confirmed by histology for the late stimulation group and the lack of infarct in the early stimulation group, suggesting that early sensory stimulation treatment is correlated with protecting the ischemic cortex by desynchronizing widespread (mesoscopic) post-ischemic pathological neuronal dynamics following pMCAo.

## Methods

All procedures were in compliance with NIH guidelines and approved by University of California, Irvine Animal Care and Use Committee (Protocol No. 1997-1608, assurance ID No. A3416.01).

### Surgical preparation

Adult (295–400 g) male Sprague Dawley rats (Charles River Laboratories, Wilmington, MA) were anesthetized by intraperitoneal injection of sodium pentobarbitol (55 mg/kg, body weight (b.w.)). Supplemental injections of sodium pentobarbital (14 mg/kg, b.w.) were given throughout the surgical preparation and electrophysiological recordings to maintain anesthesia. A self-regulating thermal blanket measured and maintained anesthetized body temperature at 37 °C. A subcutaneous injection of Dextrose (5%, 3 mL) and an intraperitoneal injection of atropine (0.05 mg/kg b.w) were administered every 6 h of experimentation.

In preparation for electrophysiological recordings, high spatial resolution functional imaging (Intrinsic Signal Optical Imaging, ISOI) was performed through a 6.5 × 8 mm imaging window of thinned skull over the left posterior medial barrel subfields (PMBSF) of the somatosensory cortex. As detailed in previous studies, ISOI captured evoked responses when a single (C2) whisker was deflected at a rate of 5 Hz for 1 s^7–9^. The C2 functional representation visualized by ISOI provided a way to position electrodes relative to PMBSF generating a reliable spatial placement of electrodes. MCA is a major provider of blood flow to somatosensory cortex^10^ and, thus, positioning electrodes in PMBSF allowed for reliable recordings of ischemic cortex after MCA occlusion.

A 1.5 mm × 5 mm craniotomy centered at the C2 whisker functional representation and a 2 mm × 2 mm craniotomy above the MCA’s M1 branch were then performed for electrode placement and pMCAo respectively.

### Surgical occlusion (pMCAo)

In the MCA craniotomy window, two surgical ligatures were inserted beneath the middle cerebral artery’s (MCA) initial (M1) segment after minimal dura removal. After surgical preparations, a fixed electrode array (refer to *Electrophysiology*) was lowered into cortex with the second recording location centered at the C2 functional representation. Electrodes settled in the brain for approximately 1 h prior to performing baseline recordings. Without setup disruption, surgical ligatures were synched, and a transection was performed between surgical knots after baseline recordings (for details see^11^).

### Sensory stimulation

In +0 h and +3 h stimulation animals, 100 baseline sensory stimulation trials were collected prior to pMCAo with a similar protocol as post pMCAo sensory stimulation. During baseline stimulation, a single whisker (C2) was deflected for 5 pulses at 5 Hz every 27s. Post-occlusion sensory stimulation involved deflecting a single whisker (C2) for 5 pulses at 5 Hz every 27s over the course of 2 h for a total of 1280 whisker deflections^2,12^. Rats were randomly assigned to experimental groups in which +0 h animals received 2 h of intermittent sensory stimulation immediately after pMCAo, +3 h stimulation animals received the same sensory stimulation treatment starting 3 h after pMCAo, and surgical sham animals were not occluded or stimulated.

### Electrophysiology

Using a fixed array of 32 microelectrodes, multi-site recordings were acquired from insulated 35 μm stainless steel wire (HML and VG bond coating insulated California Fine Wire, Grover Beach, CA). Fixed groups of four electrodes were bundled into a polyimide guide tube and adjusted to lengths of 250, 600, 1200, or 1500 μm from end of the polyimide tube comparable to Jacobs et al.’s study^9^. Placing eight guide tubes into a custom 3D printed mold resulted in a fixed 0.65 mm distance between each polyimide tube and its neighbor. Impedance of electrodes was measured prior to recordings and maintained at approximately 150 kΩ. Signals were amplified and digitized at a 22 kHz sample rate (SnR system, Alpha Omega, Nazareth, Israel) and down sampled to 2.2 kHz for analysis. Raw signals were acquired continuously for a baseline period and for the 5 h directly after the pMCAo. After sufficient baseline sampling, pMCAo occurred prior to recordings resuming during the acute (0–5 h) post-occlusion period. Raw signals were band-pass filtered for LFP (1 to 300 Hz) using a two-pole Butterworth function in MATLAB. Channels identified as noisy (9.38% of total channels) were not included in analyses. A stainless-steel ground wire was placed over the cerebellum between the skull and skin.

### Data analysis

#### Spatiotemporal synchrony

To measure neuronal synchrony, a cross-correlation matrix was derived for each electrode’s LFP relative to each other electrode’s LFP in the microelectrode array for each 1 s. For each cross-correlation comparison, one of the LFP traces in each pair was shifted up to 100 ms to account for leading and lagging comparisons. The delay at which cross-correlation was maximized was read out as the time lag. Cross-correlation analyses were repeated every (non-overlapping) 1 s of the baseline and during the acute post-pMCAo period totaling 21,600 correlations for each pair of microelectrodes. To simplify the 32x32 matrix generated for each recorded second and quantify how cross-correlations changed over the entire recording time series (see ‘Results’), all recorded electrodes pairs’ maximum cross-correlation coefficients (r^2^) were averaged for each recording second and reported as function of time. To combine average r^2^ trends across animals within an experimental group, the average r^2^ values for each subject was normalized using a z-score whose mean and standard deviation were derived from the 30-min non-stimulation baseline from the same subject.

#### Power analysis and power burst extraction

Morlet wavelet analysis, which offers increased temporal precision relative to a traditional periodogram, was also applied using in-house code to derive estimates of power for a logarithmic set of frequencies ranging from 1 to 128 Hz. Power estimates calculated by Morlet wavelet were normalized using a z-score whose mean and standard deviation were determined using the entire (30min) baseline interval.

Temporally discrete bursts of high power were then isolated in time by determining when power in a predefined frequency band consecutively exceeded a threshold for 100 or more milliseconds. The root mean square (RMS) of the Morlet wavelet-derived power for each frequency was set as the power threshold.

#### Spatiotemporal synchrony after power burst extraction

To determine the contribution of temporally discrete power bursts to spatiotemporal synchrony, LFP was scrambled in between the start and end time points of each burst identified for all frequencies in all electrodes for the entire recording period. Scrambling involved replacing the existing LFP trace during the burst with randomly generated amplitudes within the interquartile range of the LFP amplitude. Cross-correlations were then rerun on LFP that included the recorded (unchanged) LFP and epochs of scrambled LFP. The cross-correlation coefficients resulting from cross-correlations of the unchanged and the scrambled LFP traces were averaged across electrodes for every 1 s of the recording time series. In each group, 1000 bootstrap samples per subject were sampled (with replacement) in baseline and post pMCAo periods separately^13^. The mean was then found across subjects prior to formatting a test statistic of the difference between the baseline and post pMCAo for each resample. The difference between baseline and post-occlusion was considered significant if bootstrap resample 95% confidence interval (CI) did not encompass the null statistic (no difference between baseline and post-occlusion periods^14^.

### Statistical analyses

Nonparametric statistics provided a way to evaluate spatiotemporal trends given the differing lengths of baseline and post pMCAo recordings. For most of analyses involved, Kruskal-Wallis and Wilcoxon’s signed-rank tests (MATLAB functions *kruskalwallis* and *ranksum* respectively) were employed to address multiple comparisons

### Histological preparation

Histology was collected 24 h after pMCAo to measure infarct size and the relative relationship between electrodes’ locations and the infarcted area and was mostly done by a researcher blinded to the experimental group. After euthanatizing the rat, the cortex was removed by gross dissection and flattened before fixation in 4% PFA and cryoprotection in a 30% sucrose (in PBS) solution. Slicing transversely through the flattened cortex (30 μm slices) allowed for all horizontal electrode locations to be visualized throughout all cortical depths. Slices were mounted onto gelatin coated slides that were then stained with cresyl violet and cover slipped. The region of ischemic stroke was identified as the absence of staining. The area of each cortical slice and the area of the infarct within each slice were quantified using the area tool in ImageJ. The infarct volume and cortical volume was estimated for each slice by extrapolating to the 1.65 mm depth of rat whisker cortex^15^ and all slices’ approximated volumes were averaged to derive each subject’s infarct volume and percentage of infarct volume to total cortical volume.

The reporting in the manuscript follows the recommendations in the ARRIVE guidelines.

## Results

### LFP spatiotemporal synchrony increased after pMCAo but decreased back to baseline levels in the presence of early treatment (+ 0 h) sensory stimulation

**Figure 1:**
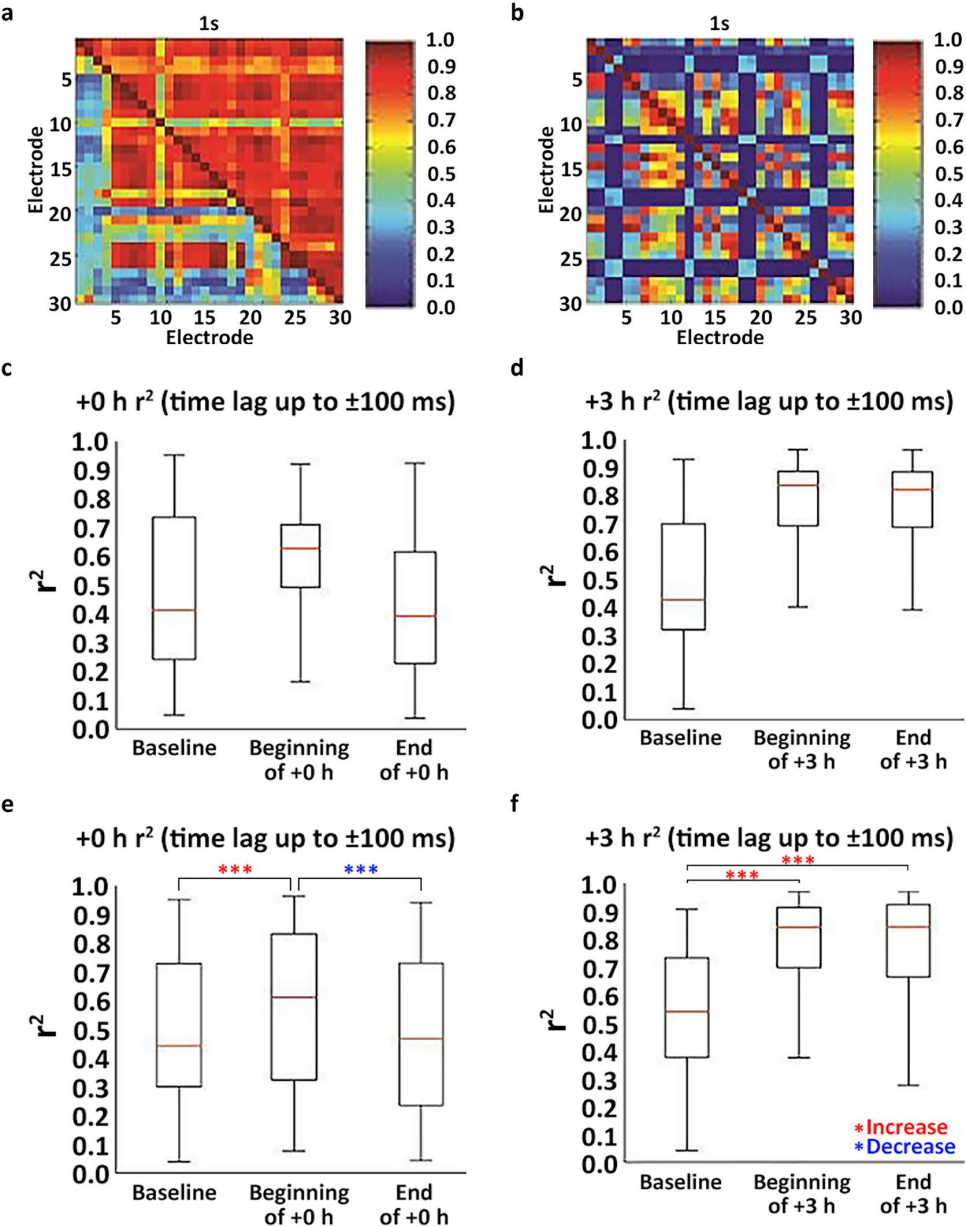
Cross-correlations demonstrated increased LFP spatiotemporal synchrony after pMCAo and differential effects of sensory stimulation treatment timing on high spatiotemporal synchrony. (**a**) Representative cross-correlograms illustrating baseline (lower left triangle) as compared to elevated post-ischemic (upper right triangle) spatiotemporal synchrony before any sensory stimulation treatment. (**b**) Representative surgical sham cross-correlograms from equivalent time points to those shown in A demonstrate typical range of cross-correlation coefficients between electrode pairs in a healthy cortex. (**c**) Representative rat’s LFP cross-correlation coefficients increase within minutes after pMCAo but decrease to near baseline levels by the end of the early + 0 h stimulation period as shown in box and whisker plots of the range of r^2^ values in a representative rat early + 0 h stimulation animal 10 min prior to pMCAo, during the first 10 min of the + 0 h (early) stimulation, and during the last 10 min of the + 0 h stimulation. (**d**) Representative rat’s LFP cross-correlation coefficients are increased after pMCAo and remain high after the late + 3 h stimulation as indicated in box and whisker plots of the range of r^2^ values in a representative the late + 3 h (late) stimulation animal 10 min prior to pMCAo, during the first 10 min of the + 3 h (late) stimulation, and during the last 10 min of the + 3 h stimulation. (**e**–**f**) Box and whisker plots of the range of cross-correlation coefficients for all + 0 h and + 3 h stimulation animals combined. Cross-correlation coefficients were significantly higher (Wilcoxon signed rank test, ***p < 0.001) directly after pMCAo as compared to baseline in both + 0 h (early) and + 3 h (late) animals. In + 0 h animals, cross-correlation coefficients significantly decreased from the beginning to the end of sensory stimulation treatment (Wilcoxon signed rank test, ***p < 0.001). The larger range of r^2^ values in e and f than c and d is likely related to between animal differences in spatiotemporal synchrony onset time after pMCAo.

**Figure 2:**
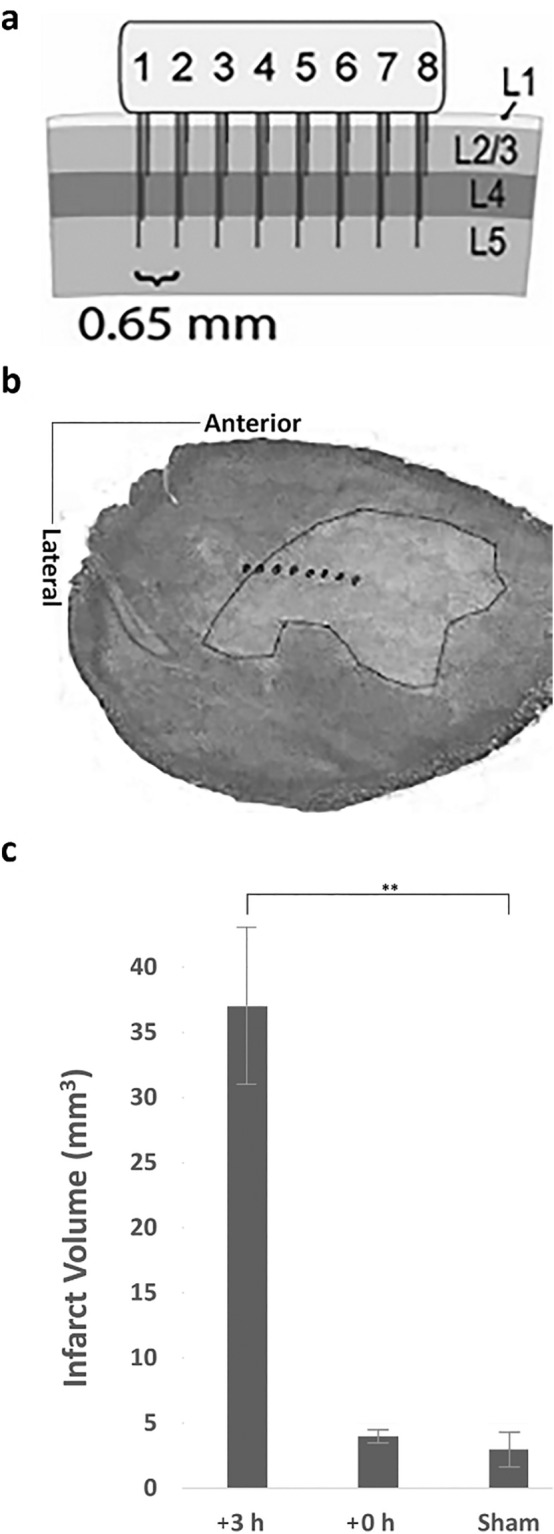
Microelectrode array and histological assessment of infarct volume. (**a**) The 32 (8 × 4) microelectrode array sampling cortical depth (**b**) Representative flattened cortical section from the late + 3 h stimulation animal in which horizontal electrodes can all be localized relative to the infarcted (bounded) region. (**c**) 24 h after pMCAo demonstrates significant cortical infarct in the late + 3 h (Wilcoxon signed rank test, **p < 0.01) but not in early + 0 h animals (Wilcoxon signed rank test, *p* = 0.61) compared to surgical sham animals. Small damage in surgical shams is due to the MCA craniotomy window and electrode placement in these animals. (**a**) and (**b**) were modified from^6^.

To determine the effects of early post-pMCAo sensory stimulation treatment on spontaneous spatiotemporal LFP dynamics, cross-correlations were calculated among all recording locations for all non-overlapping 1 s windows prior to pMCAo and in the 5 h after pMCAo including during intermittent sensory stimulation treatment following pMCAo (representative case is shown in Fig. 1a). Similar analyses were repeated in surgical sham animals (representative case is shown in Fig. 1b). Spatiotemporal synchrony assessed by cross-correlations was persistently elevated within minutes after pMCAo Fig. 1c for a representative rat and Fig. 1e for all animals combined (early stimulation, *n* = 6 rats, 95% Confidence Interval [*CI*] = 0.84, 0.92, *p* < 0.05). In early stimulation treatment, elevated post-pMCAo synchrony significantly decreased from the beginning to the end of the sensory stimulation treatment (Fig. 1c for a representative rat and Fig. 1e for all rats combined), Wilcoxon signed rank test, p<0.001). Spatiotemporal synchrony was persistently decreased after early stimulation treatment see Fig. 1c for a representative rat and Fig. 1e for all rats combined (95% Confidence Interval [*CI*] = − 0.21, − 0.15, *p* < 0.05, Bootstrapped t-statistic comparison to 0(H0)) during the 5 h post-pMCAo. These results were found to be predictive of the protection provided by early stimulation treatment as histologically observed at 24 h (Fig. 2c, infract volume for early stimulation (+0 h) 3.97 ± 0.92 mm^3^, n=6 rats, Wilcoxon signed rank test comparing early stimulation to surgical sham, n=4 rats, sham infarct volume 3.6 mm^3^ ± 1.13, *p*=0.61).

### Spontaneous LFPs spatiotemporal synchrony remains elevated during late (+ 3 h) stimulation treatment

Spatiotemporal synchrony of spontaneous LFPs was similarly evaluated in late stimulation treatment group by repeating cross-correlations during baseline and 5 h post-pMCAo. As in early stimulation treatment group, spontaneous LFPs spatiotemporal synchrony was elevated at the beginning of the post-pMCAo sensory stimulation treatment period in late stimulation animals (late stimulation, *n* = 6 *rats*, Fig. 1d for a representative rat and 1f for all rats combined, Wilcoxon signed rank test, *p* < 0.001). In contrast to the early stimulation group, late stimulation animals continued to have significantly high cross-correlation coefficients at the end of the sensory stimulation treatment period (Fig. 1d for a representative rat and 1f for all rats combined, Wilcoxon signed rank test, *p* < 0.001). The elevated LFPs spatiotemporal synchrony persisted throughout the entire post pMCAo period (95% Confidence Interval [*CI*] = 3.23, 3.34, *p* < 0.05), consistent with the significantly larger infarct of late treatment as compared to early treatment stimulation animals (Fig. 2b, c, +3 h stimulation *n* = 6 *rats*; infarct volume 36.01 ± 10.27 mm^3^, Wilcoxon signed rank test comparing late stimulation (+3 h) to sham surgery group (sham), n =4 rats, infarct volume 3.6 mm^3^ ± 1.13, *p* < 0.01). These findings are similar to our previous findings using the same experimental rat groups under the same anesthesia.

### Intermittent low multi-frequency bursts underlie spontaneous LFPs synchrony.

**Figure 3:**
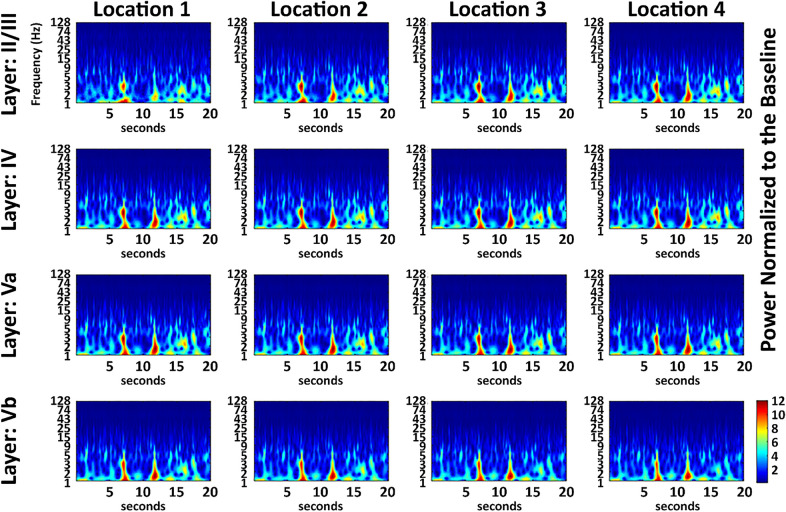
Post pMCAo bursting is recorded in all electrodes at similar time points and frequencies between electrodes. A normalized representative case from the early + 0 h sensory stimulation treatment period 1 h following pMCAo. Location 2 is centered at the peak evoked recording location and only the 16 electrodes closest to the peak recording location are shown. In the 16 electrodes shown, bursting occurred concurrently.

**Figure 4:**
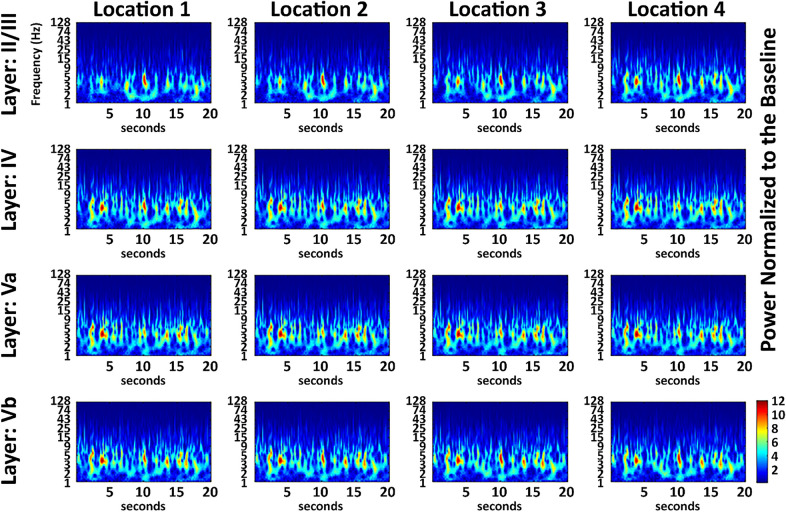
Post pMCAo bursting is recorded in all electrodes at similar time points and frequencies between electrodes. A normalized representative case from the late + 3 h sensory stimulation treatment period 1 h following the beginning of the + 3 h stimulation (i.e., 4 h following pMCAo). Location 2 is centered at the peak evoked recording location and only the 16 electrodes closest to the peak recording location are shown. In the 16 electrodes shown, bursting occurred concurrently.

Morlet wavelet analysis was applied to characterize the time-varying frequency structure of pre- and post-ischemic periods. Temporally discrete increases (bursts) in power occurred across multiple frequencies and were particularly robust, withstanding baseline normalization after pMCAo (Figs. 3, 4). The increase in spontaneous low frequency power bursts (including delta (1–4 Hz), theta (4–8 Hz) and alpha (8–12 Hz) bands) persisted throughout the post pMCAo period in the late (+3 h) stimulation group. Observed power bursts occurred across all recording electrodes coordinated in time as seen 1 h after the beginning of stimulation in the early (+0 h) (Fig. 3) and late (+3 h) stimulation (Fig. 4).

**Figure 5:**
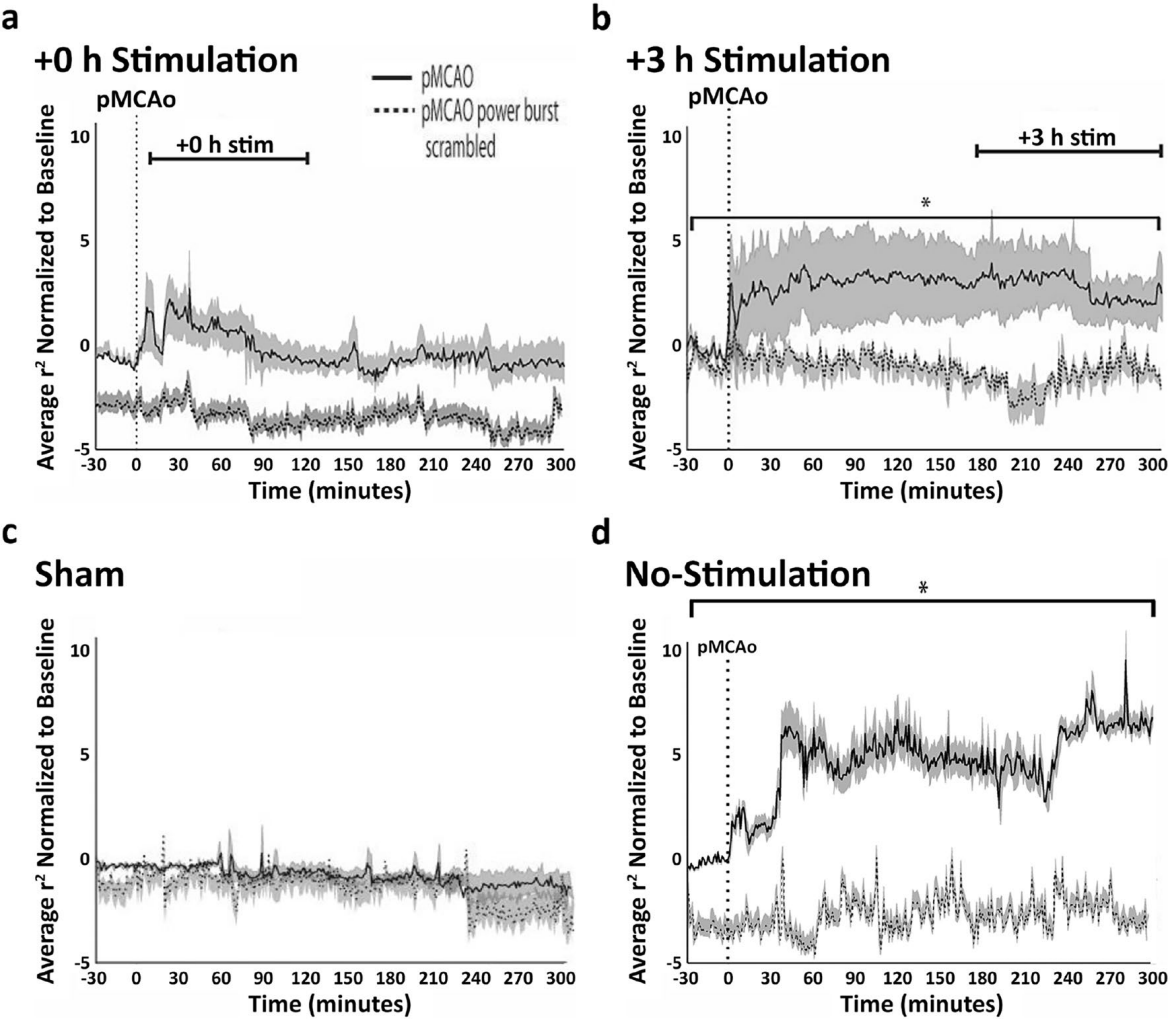
Multi low frequency bursts underlie spontaneous LFP synchrony. (**a**) The entire early + 0 h group. (**b**) The entire late + 3 h group, (**c**) the entire sham group, and (**d**) the entire no-stimulation control group. The solid black line in all panels represent the average cross-correlation coefficients normalized to baseline, gray surround denotes SEM. The dashed black line in all panels show the same analysis when ran on LFP containing scrambled time periods (when power bursting was identified) and unchanged (non-power bursting) time periods. Note how in both groups the scrambled cross-correlations results remain at pre-pMCAo baseline level. Groups (**c**) and (**d**) were modified from reference^6^.

To determine the effect of power bursting time periods on elevated post-ischemic LFP spatiotemporal synchrony, temporally discrete increases in power were isolated and were used to selectively scramble LFP signal only during bursting time periods (see ‘Methods’). When repeating cross-correlation analyses on LFPs with scrambled bursts, cross-correlation coefficients did not increase after pMCAo either for the entire early (+ 0 h) treatment group or the entire late (+ 3 h) group (Fig. 5b, the cases of 95% Confidence Interval [*CI*] =  − 0.05, 0.04, *p* > 0.05; Bootstrapped t-statistic comparison to 0(H0)). The difference between the scrambled and non-scrambled lines at baseline (Fig. 5a) is potentially due to the existence of shorter than our criterion (< 100 ms) bursts in the non-scrambled (upper) trace. These figures should be compared to the cases of the entire sham group (Fig. 5c) and to the entire no-stimulation control group (Fig. 5d, n = 7 rats). Notably, the initial increase in LFP synchrony can be seen in all groups except the sham group, an observation that entails a robust finding as it is seen in three different experimental groups.

## Discussion

The relevance of neuronal activity dynamics during ischemia has not received much attention despite the established association between assessments of neural function and improved stroke outcome prediction^16–19^, (see^20^ for an exception). The protective effect of neuronal activity evoked by early stimulation also demonstrates a pivotal need to understand the potential relationship between evoked neuronal activity and ischemic outcome^2,21–24^. We were especially interested in whether widespread, mesoscopic level neuronal states during the 5 h hyperacute period after pMCAo could distinguish between protective early treatment and damaging late treatment sensory stimulation groups given the clear histological outcome discrepancies, outcomes which are consistent with our previous results in both treatment groups^2,21,22,25^.

We have previously demonstrated in a no-stimulation control group that spontaneous LFP spatiotemporal synchrony is elevated within minutes after pMCAo and remains high throughout the acute post-ischemic 5 h period whereas sham-surgery controls didn’t show such increase^6^. In the current study, we found the same results regarding increase after pMCAo but that post-pMCAo spatiotemporal synchrony was only persistently high in animals incurring infarct (late stimulation) but not in protected (early stimulation) animals. Furthermore, in early treatment stimulation animals, spatiotemporal synchrony increased within minutes after pMCAo and the elevated spatiotemporal synchrony decreased back to pre-pMCAo level baseline over the course of early intermittent sensory stimulation treatment. The gradual dissipation of high spatiotemporal synchrony over the early stimulation treatment period is temporarily consistent with two separate processes that could be linked: (1) the reduction in synchrony of the underlying multi-frequency oscillatory intermittent bursts, and (2) the slow recovery profile of residual blood flow within the permanently occluded MCA during early stimulation. Early stimulation treatment is known to augment protective retrograde blood flow within the occluded MCA via collateral vasculature providing an alternative source of reperfusion necessary for the observed neuroprotection in our pMCAo model^2,21,26^. Therefore, in our pMCAo model it seems that the retrograde collateral flow, despite its low velocity and flux values^26^ is still sufficient to sustain evoked neuronal activity in the ischemic territory even 5 h after pMCAo^6^. The role of collateral vasculature in our model is consistent with human collateral therapeutic strategies that focus on recruiting and stabilizing collateral blood flow after ischemic onset to offset potential injury^27–29^. Future studies should simultaneously assess collateral blood flow dynamics and spontaneous LFP dynamics to further examine their relationship. However, other mechanisms could also be involved in the interaction between increased LFP synchrony and infarct. It is possible that the role of protective stimulation is also in enhancing astrocytes to support neuronal energetics through the astrocyte-to-neuron lactate shuttle—a mechanism that is currently being investigated in our lab—that fails without the presence of protective sensory stimulation. However, we would also like to emphasize that there is a chicken-egg issue regarding the correlative nature of our findings: is it that the growing presence of synchronized LFP somehow causes the ischemic cortex to infarct, or that increasing ischemic damage causes increased LFP synchrony?

Our current and previous^6^ results using the microelectrode array highlight the unexpected importance of widespread spontaneous LFPs dynamics. Spontaneous LFPs dynamics uniquely provide information on the pathological state of the cortex following pMCAo that is unavailable from evoked LFP or evoked MUA, which are the traditional measures of cortical health. Indeed, whereas spontaneous LFPs became spatiotemporally synchronized within few minutes following pMCAo, evoked LFPs remained mostly similar to pre-pMCAo baseline for 3 h and started to change only at 3–5 h post pMCAo recording session whereas evoked MUA didn’t change^6^. These findings demonstrate that neurons in the ischemic area remain mostly intact in their ability to respond to sensory stimulation for few hours following dorsal pMCAo despite major widespread changes in spatiotemporal synchrony of their spontaneous LFPs immediately after pMCAo, suggesting that the two processes are likely uncoupled. However, these processes (evoked LFP, MUA vs. spontaneous LFP) are not completely uncoupled, at least for the early post-pMCAo period (2 h following pMCAo), as the early stimulation evoked activity in the ischemic territory results in a clear desynchronizing of spontaneous LFPs by desynchronizing the underlying low multi-frequency intermittent bursts. Our findings on the protective effects of sensory stimulation are consistent with recent findings regarding the protective effects of early external non-invasive stimulation (e.g., focused ultrasound^30^, and tDCS^31–33^ in a MCAo model. Taken together, the importance of neuronal excitability dynamics and the presence of sensitive protective and destructive periods in hyperacute phase of stroke recovery can now be generalized with their presences in other phases of stroke recovery beyond the hyperacute state (acute, subacute, chronic states)^34^. We would like to emphasize that our current findings are only relevant to the hyperacute state of the ischemic cortex.

The distance between the first and last microelectrode is 4.5 mm and the array recorded at up to a cortical depth of 1500 μm (Fig. 2a). How can a single whisker intermittent stimulation administrated within the first 2 h following pMCAo protect such a large cortical volume from impending stroke damage? Employing functional imaging and post-imaging neuronal recordings, we have repeatedly demonstrated in the rat that single whisker stimulation activates a very large cortical area at the evoked LFP level^8,9,15,35^ see review^36^. Supporting and further extending these findings by employing the same microelectrode array before and after pMCAo, we have recently quantified again the spread of the evoked LFPs negative amplitude as it continuously and progressively decays over cortical distance away from peak evoked location. We demonstrated that the LFP negative peak amplitude measured at the last microelectrode at a distance of 3.9 mm away from peak activation (peak activation recorded at the second electrode group; see Figs. 3, 4) during pre-pMCAo baseline was still at 20% amplitude of peak evoked activity. Two hours post-pMCAo it remained at 17% of peak evoked response^6^. These findings imply that the evoked LFPs spread is even larger than 3.9 mm away from peak activation as it has not fully decayed at this distance. Indeed, these findings are consistent with our repeated demonstrations of the existence of a system of horizontal axonal projection in cortical gray matter that include extremely long-range projections (up to 5mm), a system that supports the widespread evoked LFPs spread over cortical territory^15,37–39^ see also review^36^. Therefore, it is likely that the widespread LFPs evoked by single whisker stimulation encompasses most if not the entire ischemic cortical volume characterized by spontaneous LFP spatiotemporal synchrony. Such spread therefore is potentially capable of desynchronizing the underlying bursts in the ischemic territory. The inability of the identical whisker stimulation delivered 3 h after pMCAo to protect the cortex despite having a similar widespread evoked LFP (18% peak negative amplitude at the last electrode 3–5 h following pMCAo^6^) replicates our previous findings regarding the lack of protection and even exacerbation of stroke damage (as compared with no-stimulus controls damage) by the same intermittent whisker stimulation at 3 h post pMCAo^2,21,22,25^, as seen in Fig. 2c.

In summary evaluating widespread synchronization of spontaneous neuronal activity and its underlying oscillatory dynamics that evolve after pMCAo is an important clue for understanding the neurophysiological effects of sensory stimulation treatment and the time window of efficacious sensory stimulation. Due to the LFP-EEG connection, identifying large-scale electrophysiological signatures of acute ischemia may be translationally relevant if our findings can be applied to guide EEG analyses, because measures of neuronal function during stroke are known to augment injury-based diagnostics^19,40^. Indeed, recent studies in humans with stroke using EEG data recorded in the emergency department show that broad frequency ranges of EEG (theta, alpha, and beta) can be used with machine learning algorithms to distinguish stroke patients from other patients who present similar symptoms ("possible" stroke) in the ER^41^, suggesting EEG is a potential diagnostic tool suitable for utilization in prehospital triage of stroke patients^42^.
